# Supplementary material for: Differential Methylation of Telomere-Related Genes Is Associated with Kidney Disease in Individuals with Type 1 Diabetes
Source: Genes (Basel). 2023 Apr 30;14(5):1029. doi: 10.3390/genes14051029 (PMC10217816; doi:10.3390/genes14051029)
Supplement: Supplementary file 1 [file genes-14-01029-s001.zip › genes-2253974-supplementary/Supp_figures.pdf]

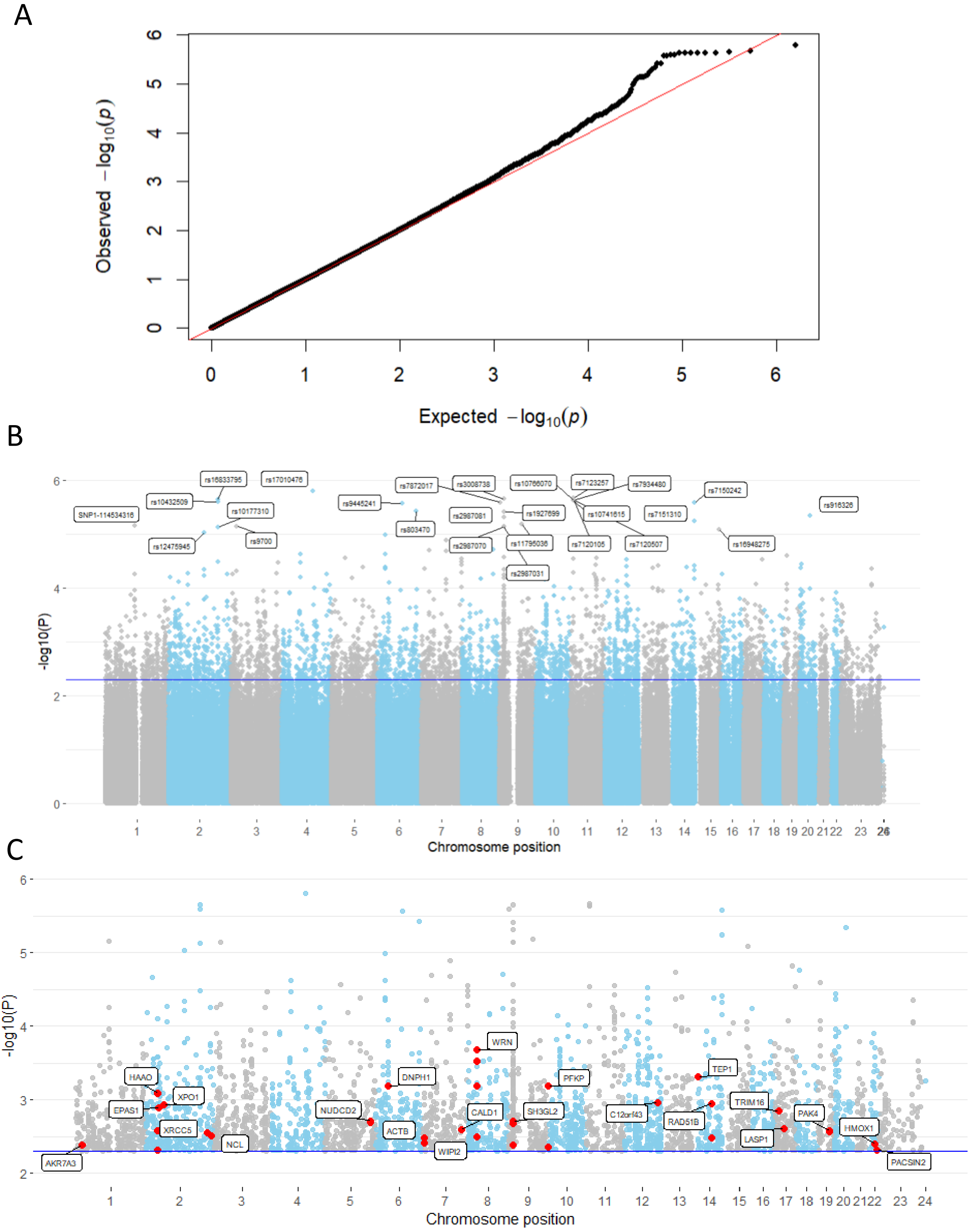

Figure S1
Hill and Duffy *et al*, 2023

A

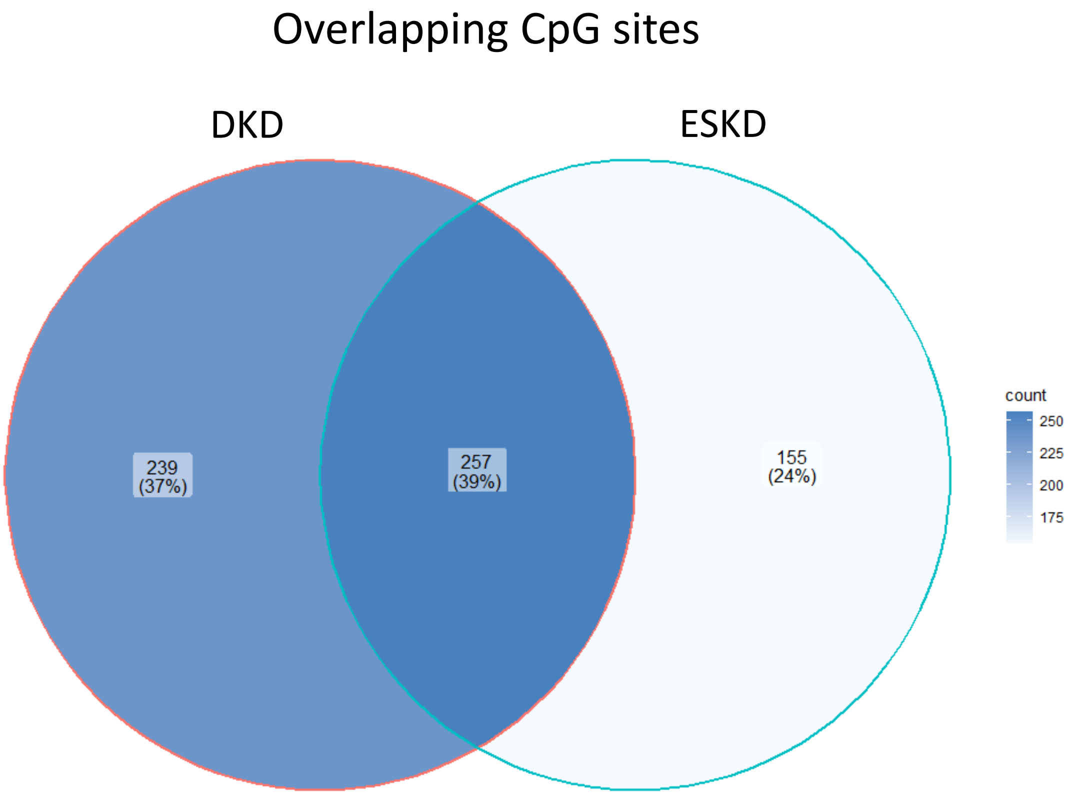

B

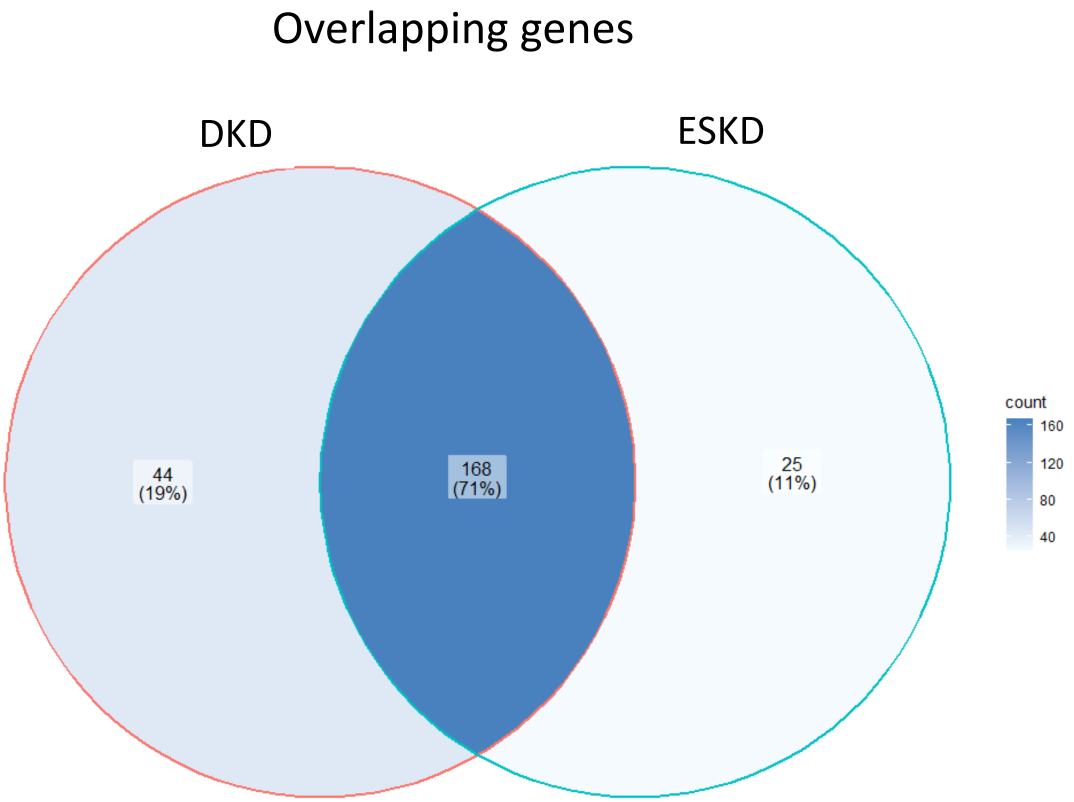

Figure S2
Hill and Duffy *et al*, 2023

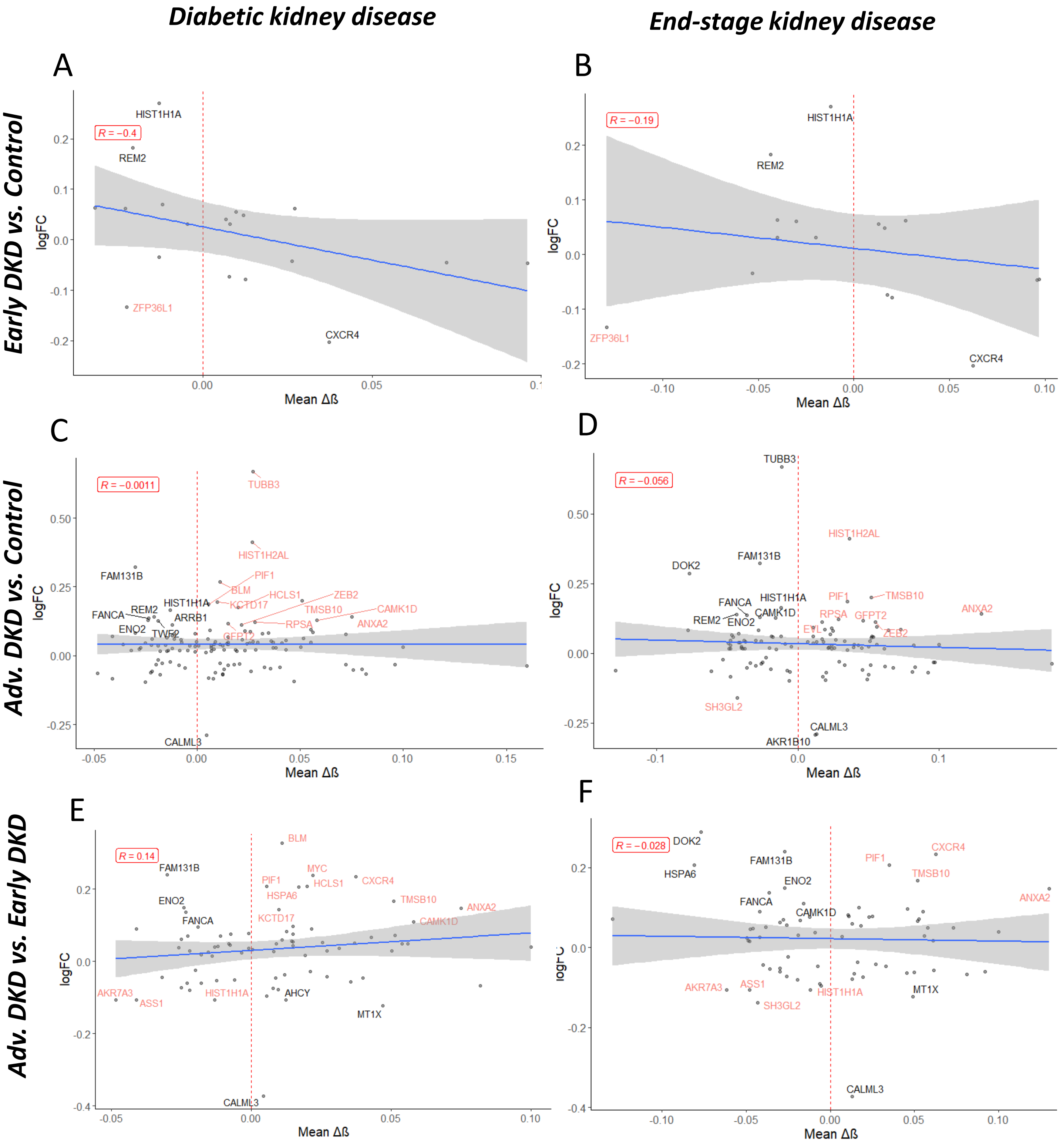

Figure S3
Hill and Duffy *et al*, 2023

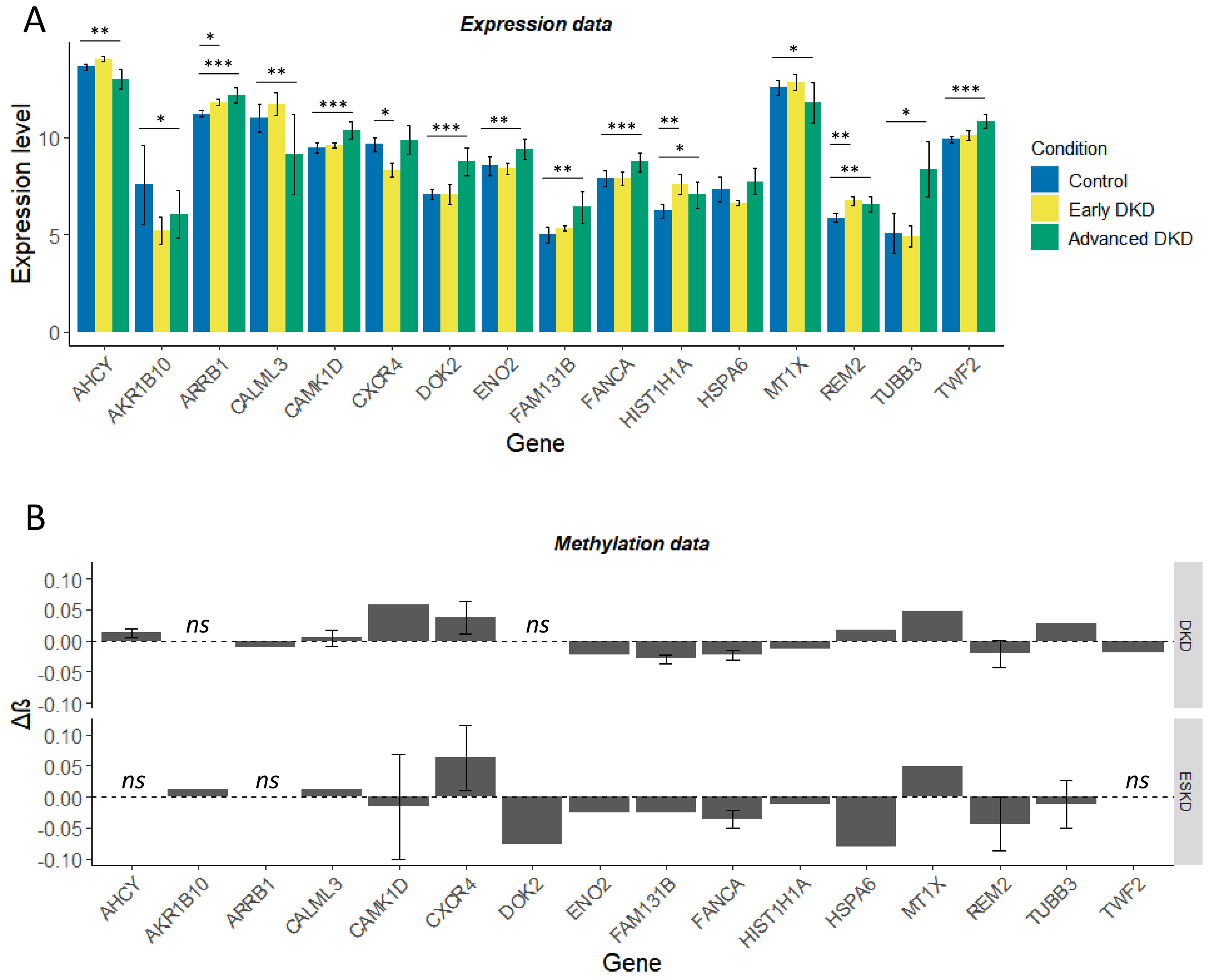

Figure S4
Hill and Duffy *et al*, 2023

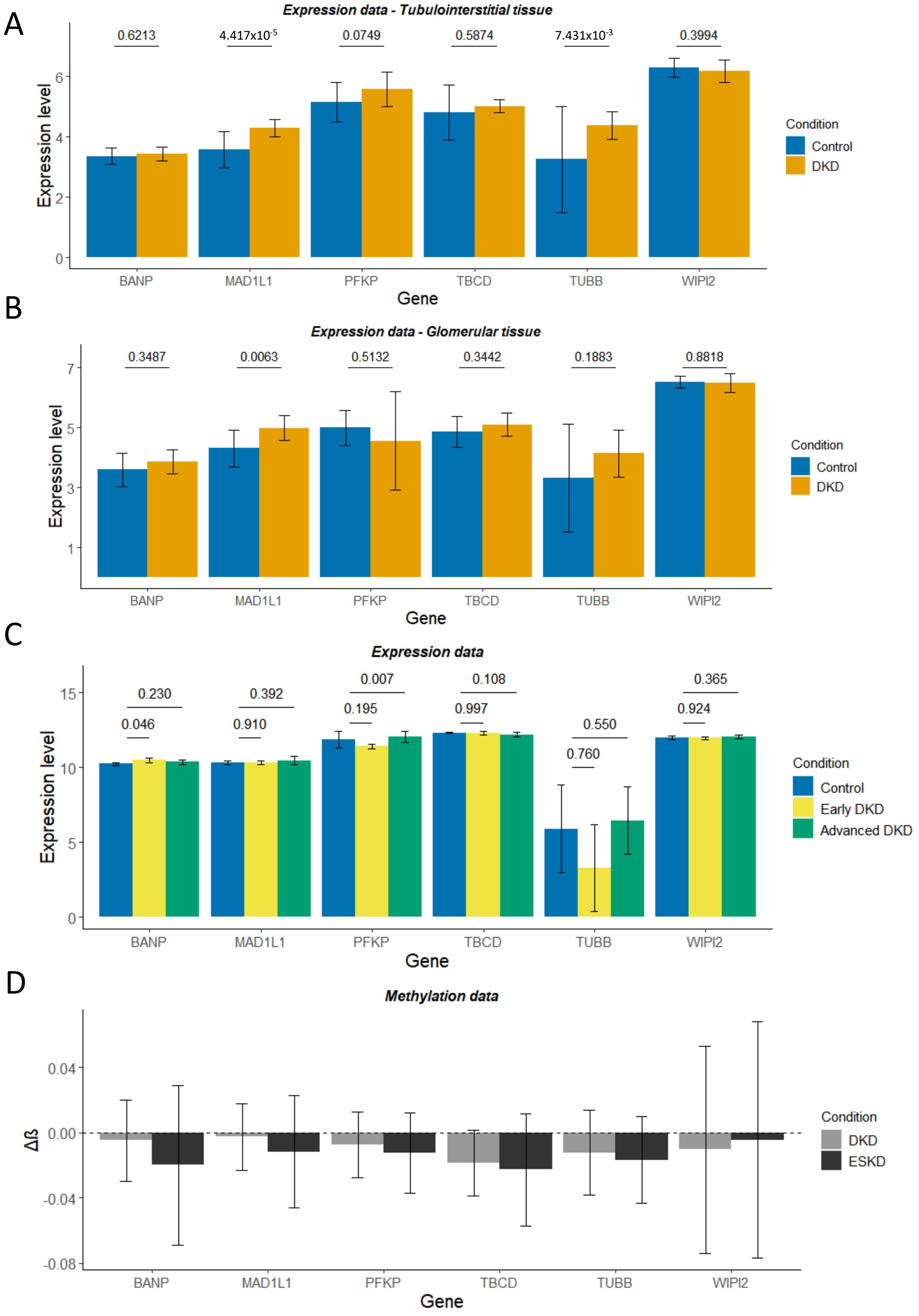

Figure S5
Hill and Duffy *et al*, 2023

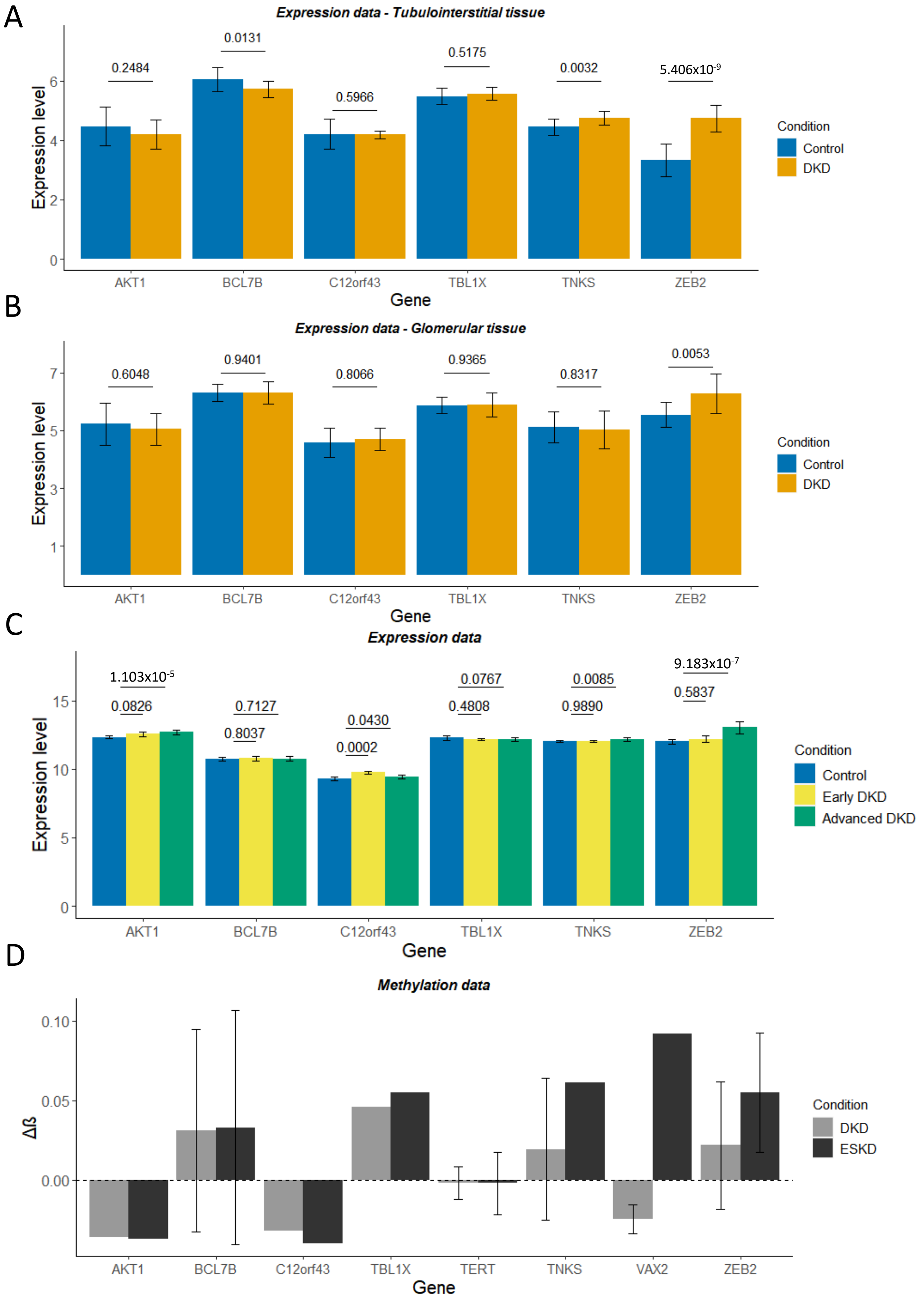

Figure S6

Hill and Duffy *et al*, 2023
