# Supplementary material for: Differential Methylation of Telomere-Related Genes Is Associated with Kidney Disease in Individuals with Type 1 Diabetes
Source: Genes (Basel). 2023 Apr 30;14(5):1029. doi: 10.3390/genes14051029 (PMC10217816; doi:10.3390/genes14051029)
Supplement: Supplementary file 1 [file genes-14-01029-s001.zip › genes-2253974-supplementary/Telomere_dkd_Supp_Materials_2023.pdf]

Supplementary Materials

Contents

Supplementary Methods 1

Study Cohorts 1

Telomere length determination 2

Genome wide association study 2

Mendelian Randomisation 3

Gene ontology analysis 3

Differential expression analysis 3

Supplementary References 4

Supplementary Methods

Study Cohorts

All Ireland-Warren 3-Genetics of Kidneys in Diabetes UK Collection (UK-ROI) [1]: All participants (n=1,726) were recruited for the All-Ireland, Warren 3, and Genetics of Kidneys in Diabetes (GoKinD) UK collections and defined as self-reported White individuals from the British isles with T1D diagnosed before the age of 31. Cases were defined as individuals with T1D with persistent proteinuria (>500 mg/24hr) developing over 10 years after the diagnosis of diabetes, hypertension (≥135/85 mmHg) and retinopathy. Controls were defined by having T1D for at least 15 years but had no evidence of microalbuminuria on repeated testing and were not receiving antihypertensive medication. ESKD was defined as individuals requiring chronic dialysis or have received a kidney transplant [2].

Danish Steno Diabetes Center (Steno) [3]: Participants were recruited in Denmark with DKD cases being defined as persistent albuminuria (≥300 mg/24 h) in two of three consecutive 24 h urine collections, presence of retinopathy and no evidence of other renal or urinary tract disease. Controls were defined as having longstanding T1D (more than 15 years) and a persistent urinary albumin excretion rate (AER) within the normal range (<30 mg/24 h).[4] Telomere length (assessed by qPCR) was determined in 78 cases and 153 controls from this cohort. Telomere length for this cohort was previously determined via Southern Blotting [5]. All included subjects were of Caucasian descent.

Finnish Diabetic Nephropathy Study (FinnDiane)[6]: A collection consisting of participants (n=2,910) of Finnish descent with T1D diagnosed before the age of 35 with insulin treatment beginning within 1 year of diagnosis. Disease status (control, microalbuminuria AND macroalbuminuria) WAS defined by AER or albumin creatinine ratio (ACR), as described previously [2]. ESKD was defined as ongoing dialysis or kidney transplant. Control patients had T1D for longer than 15 years, with normal AER.

Genetics of Kidneys in Diabetes US Study (US GoKinD) [7]: Consists of individuals (n=1,595) diagnosed with T1D before the age of 31 who began insulin treatment within 1 year of diagnosis. Cases were defined as individuals aged between 18-54 years with T1D for at least 10 years and DKD. The DKD definition includes individuals with ESRD, dialysis or kidney transplant and persistent macroalbuminuria (at least 2 out of 3 tests positive for albuminuria by dipstick (>1+), or ACR >300 mg albumin/mg of urine creatinine. Controls were diabetic patients with at least 15 years of T1D, aged 18-59, with no evidence of DKD; individuals recruited to the control group were based on the same inclusion criteria as UK-ROI [2]. Analysis of the U.S. GoKinD cohort was limited to individuals whose self-reported primary ethnicity was white.

Telomere length determination

Telomere length values were determined via monochrome quantitative polymerase chain reaction (qPCR) using relative ratio of telomere repeat copy number to a single copy gene 36B4,[8] in DNA derived from whole blood. PCR reactions were performed in triplicate using 384-well PCR plates with 10 ng of genomic DNA template, 5 µL of 2x LightCycler® 480 SYBR Green I Master mix and primer pairs specific to each reaction at a final concentration of 300 nm. Primer pair sequences for the telomere PCR were (5′ to 3′): Telo1 CGGTTTGTTTGGGTTTGGGTTTGGGTTTGGGTTTGGGTT and Telo2 GGCTTGCCTTACCCTTACCCTTACCCTTACCCTTACCCT. Primer pair sequences for the 36B4 PCR were (5′ to 3′): 36B4d CCCATTCTATCATCAACGGGTACAA and 36B4u CAGCAAGTGGGAAGGTGTAAT CC. A reference DNA sample was used to generate a standard curve that was run in triplicate. Thermal cycling was performed on the 7900 Real-Time PCR System (Applied Biosystems, California, United States) with the following conditions: Hold for 2 minutes at 50°C; 10 minutes at 95°C; 40 cycles of 15 seconds at 95°C, 1 minute at 60°C; 15 seconds at 95°C; 1 minute at 60°C; 30 seconds at 95°C; and 15 seconds at 60°C. SDS V2.4.1 (Applied Biosystems) software was used to determine values for both the telomere and 36B4 for each sample using a standard curve. Results were evaluated using IBM SPSS Statistics 21 (SPSS Inc. Chicago, IL) and R studio (v. 1.4.1717), R version 4.1.1 [9]. Telomere length of 391 of these samples was previously determined via Southern Blot [5]. Martin-Ruiz *et al.* have compared qPCR and Southern Blot methods, with both methods shown to be capable of determining similar measurements of relative telomere length [10,11].

Genome wide association study

A literature search was conducted to identify genes associated with telomere maintenance and function using PubMed (www.ncbi.nlm.gov/pubmed). Relevant gene names were revised to those approved by the HUGO Gene Nomenclature Committee [12]. For the GWA analysis of DKD and ESKD, 749,173 and 746,125 SNPs were analysed after QC, respectively. For the GWA analysis assessing relative telomere length (RTL) in the UK-ROI population, 791,687 directly typed SNPs were analysed. The RTL GWA analysis was carried out via PLINK (v. 1.9), using age as a covariate [13]. Genetic variants nominally associated with RTL in this analysis were assigned to genes using the online Variant Effect Predictor (VEP) tool (GRCh37 assembly) [14]. QQ and Manhattan plots were created via the R package qqman (v. 0.1.8) in R studio (v. 1.4.1717) [9,15].

Mendelian Randomisation

Two Sample Mendelian randomisation was performed consistent with methods described by Park *et al*. [16], with the additional removal of overlapping SNPs *via* linkage disequilibrium clumping and the removal of SNPs with flipped alleles for clarity [17]. Codd *et al.* identified a genetic instrument for leukocyte telomere length harnessing 472,174 individuals from a UK Biobank cohort [18]. The 130 SNPs identified by Codd *et al.* were used as instrumental variables (IVs) in this T1D cohort. Separately, 33 SNPs harnessed by Park *et al.* were utilised as an additional set of IVs [16]. Park *et al.* derived these SNPs from the 52 SNPs associated with telomere length originally identified by Li *et al.*, removing non-overlapping and monoallelic SNPs, as well as SNPs with a significant association with phenotypes other than telomere length, together with a relatively weak association with telomere length to reduce potential pleotropic effects [16,19]. Beta values for the Park *et al.* instrumental variables represent telomere attrition. Beta values for the Codd *et al.* instrumental variables represent telomere length. Outcome data for each SNP of the genetic instrument was extracted from the GEnetics of Nephropathy an International Effort (GENIE) meta-analysis (dbGaP Study Accession: phs000389.v1.p1), considering either DKD or ESKD as the phenotype. Analyses were carried out in R Studio (v. 1.4.1717) using the TwoSampleMR R package (v. 0.5.6, default settings, methods: mr_ivw, mr_simple_median, mr_weighted_median and mr_egger_regression, run_mr_presso()), except for the MR RAPS analyses, which were carried out using the mr.raps package directly (v. 0.2, default settings) [9,20–22].

Gene ontology analysis

The 376 telomere-related genes identified in this analysis were used as the background gene set. DKD and ESKD gene sets were those genes found to be significantly differentially methylated in the respective DKD or ESKD EWA analyses. Gene names were converted to their relevant Enterez IDs using the online DAVID ID conversion tool [23–25]. The ViSEAGO Enterez annotations (ID: 9606) were harnessed. Processes were implemented as described by Brionne *et al.* [26]. The minimum number of annotated genes per GO term (nodeSize) was set to 5. The classic algorithm was harnessed, together with the Fisher statistic, utilising a significance cut off of $P<0.01$. Semantic similarity measures were computed using the 'Wang' distance parameter and the heatmap aggregation criteria was 'ward.D2' (Partitioning Around Medoids (pam)Stage=TRUE, pamRespectsDendro=TRUE, deepSplit=2, minClusterSize =2).

Differential expression analysis

Gene expression data from previous RNA-sequencing analyses of DKD and control tissues was utilised in the limma differential expression analyses (Galaxy Version 3.48.0+galaxy1) on the Galaxy web platform (www.usegalaxy.org) [27–31]. The limma-voom method was harnessed for analyses using both datasets. P-adjusted values were calculated via the "Benjamini and Hochberg (1995)" method, the default limma-voom option [32]. For the raw read counts provided by Levin *et al.*, genes without more than 2 counts in at least 31 out of 39 samples were filtered out and TMM was used to normalise library sizes. For the normalised read counts provided by Fan *et al.*, no further filtering or normalisation was performed. Mean delta-beta values were determined for genes significantly associated with DKD or ESKD by averaging the delta-beta values across significant CpG sites for each gene. Correlation analysis

was performed in R studio (v. 1.4.1717) using ggplot2 (v. 3.3.5, geom_smooth, method='lm') [9,33].

Supplementary References

1. McKnight, A.J.; Patterson, C.C.; Pettigrew, K.A.; Savage, D.A.; Kilner, J.; Murphy, M.; Sadlier, D.; Maxwell, A.P. A GREM1 Gene Variant Associates with Diabetic Nephropathy. *J. Am. Soc. Nephrol.* **2010**, *21*, 773–781, doi:10.1681/ASN.2009070773.

2. Sandholm, N.; Salem, R.M.; McKnight, A.J.; Brennan, E.P.; Forsblom, C.; Isakova, T.; McKay, G.J.; Williams, W.W.; Sadlier, D.M.; Mäkinen, V.P.; et al. New Susceptibility Loci Associated with Kidney Disease in Type 1 Diabetes. *PLoS Genet.* **2012**, *8*, e1002921, doi:10.1371/journal.pgen.1002921.

3. Tarnow, L.; Groop, P.H.; Hadjadj, S.; Kazeem, G.; Cambien, F.; Marre, M.; Forsblom, C.; Parving, H.H.; Trégouët, D.; Thévard, A.; et al. European Rational Approach for the Genetics of Diabetic Complications - EURAGEDIC: Patient Populations and Strategy. *Nephrol. Dial. Transplant.* **2008**, *23*, 161–168, doi:10.1093/ndt/gfm501.

4. Lajer, M.; Tarnow, L.; Fleckner, J.; Hansen, B. V.; Edwards, D.G.; Parving, H.H.; Boel, E. Association of Aldose Reductase Gene Z+2 Polymorphism with Reduced Susceptibility to Diabetic Nephropathy in Caucasian Type 1 Diabetic Patients. *Diabet. Med.* **2004**, *21*, 867–873, doi:10.1111/j.1464-5491.2004.01259.x.

5. Astrup, A.S.; Tarnow, L.; Jorsal, A.; Lajer, M.; Nzietchueng, R.; Benetos, A.; Rossing, P.; Parving, H.H. Telomere Length Predicts All-Cause Mortality in Patients with Type 1 Diabetes. *Diabetologia* **2010**, *53*, 45–48, doi:10.1007/s00125-009-1542-1.

6. Syreeni, A.; El-Osta, A.; Forsblom, C.; Sandholm, N.; Parkkonen, M.; Tarnow, L.; Parving, H.H.; McKnight, A.J.; Maxwell, A.P.; Cooper, M.E.; et al. Genetic Examination of SETD7 and SUV39H1/H2 Methyltransferases and the Risk of Diabetes Complications in Patients with Type 1 Diabetes. *Diabetes* **2011**, *60*, 3073–3080, doi:10.2337/db11-0073.

7. Williams, W.W.; Salem, R.M.; McKnight, A.J.; Sandholm, N.; Forsblom, C.; Taylor, A.; Guiducci, C.; McAteer, J.B.; McKay, G.J.; Isakova, T.; et al. Association Testing of Previously Reported Variants in a Large Case-Control Meta-Analysis of Diabetic Nephropathy. *Diabetes* **2012**, *61*, 2187–2194, doi:10.2337/db11-0751.

8. Cawthon, R.M. Telomere Measurement by Quantitative PCR. *Nucleic Acids Res.* **2002**, *30*, 1–6, doi:10.1093/nar/30.10.e47.

9. RStudio Team RStudio: Integrated Development Environment for R 2020.

10. Martin-Ruiz, C.M.; Baird, D.; Roger, L.; Boukamp, P.; Krunic, D.; Cawthon, R.; Dokter, M.M.; Van der Harst, P.; Bekaert, S.; De Meyer, T.; et al. Reproducibility of Telomere Length Assessment: An International Collaborative Study. *Int. J. Epidemiol.* **2015**, *44*, 1673–1683, doi:10.1093/ije/dyu191.

11. Martin-Ruiz, C.M.; Baird, D.; Roger, L.; Boukamp, P.; Krunic, D.; Cawthon, R.; Dokter, M.M.; Van Der Harst, P.; Bekaert, S.; De Meyer, T.; et al. Is Southern Blotting Necessary to Measure Telomere Length Reproducibly? Authors' Response to:

Commentary: The Reliability of Telomere Length Measurements. *Int. J. Epidemiol.* **2015**, *44*, 1686–1687, doi:10.1093/ije/dyv169.

12. Gray, K.; Daugherty, L.; Gordon, S.; Seal, R.; Wright, M.; Bruford, E. Genenames.Org: The HGNC Resources in 2013. *Nucleic Acids Res.* **2012**, *41*, D545-52, doi:10.1093/nar/gks1066.

13. Purcell, S.; Neale, B.; Todd-Brown, K.; Thomas, L.; Ferreira, M.A.R.; Bender, D.; Maller, J.; Sklar, P.; De Bakker, P.I.W.; Daly, M.J.; et al. PLINK: A Tool Set for Whole-Genome Association and Population-Based Linkage Analyses. *Am. J. Hum. Genet.* **2007**, *81*, 559–575, doi:10.1086/519795.

14. McLaren, W.; Gil, L.; Hunt, S.E.; Riat, H.S.; Ritchie, G.R.S.; Thormann, A.; Flicek, P.; Cunningham, F. The Ensembl Variant Effect Predictor. *Genome Biol.* **2016**, *17*, 122, doi:10.1186/s13059-016-0974-4.

15. Turner, S. Qqman: An R Package for Visualizing GWAS Results Using Q-Q and Manhattan Plots. *J. Open Source Softw.* **2018**, doi:10.21105/joss.00731.

16. Park, S.; Lee, S.; Kim, Y.; Cho, S.; Kim, K.; Kim, Y.C.; Han, S.S.; Lee, H.; Lee, J.P.; Joo, K.W.; et al. A Mendelian Randomization Study Found Causal Linkage between Telomere Attrition and Chronic Kidney Disease. *Kidney Int.* **2021**, *100*, 1063–1070, doi:10.1016/j.kint.2021.06.041.

17. Rayner, W. Genotyping Chips Strand and Build Files Available online: https://www.well.ox.ac.uk/~wrayner/strand/.

18. Codd, V.; Wang, Q.; Allara, E.; Musicha, C.; Kaptoge, S.; Stoma, S.; Jiang, T.; Hamby, S.E.; Braund, P.S.; Bountziouka, V.; et al. Polygenic Basis and Biomedical Consequences of Telomere Length Variation. *Nat. Genet.* **2021**, *53*, 1425–1433, doi:10.1038/s41588-021-00944-6.

19. Li, C.; Stoma, S.; Lotta, L.A.; Warner, S.; Albrecht, E.; Allione, A.; Arp, P.P.; Broer, L.; Buxton, J.L.; Da Silva Couto Alves, A.; et al. Genome-Wide Association Analysis in Humans Links Nucleotide Metabolism to Leukocyte Telomere Length. *Am. J. Hum. Genet.* **2020**, *106*, 389–404, doi:10.1016/j.ajhg.2020.02.006.

20. Hemani, G.; Zheng, J.; Elsworth, B.; Wade, K.H.; Haberland, V.; Baird, D.; Laurin, C.; Burgess, S.; Bowden, J.; Langdon, R.; et al. The MR-Base Platform Supports Systematic Causal Inference across the Human Phenome. *Elife* **2018**, *7*, e34408, doi:10.7554/eLife.34408.

21. Hemani, G.; Tilling, K.; Smith, G.D. Orienting the Causal Relationship between Imprecisely Measured Traits Using Genetic Instruments. *PLoS Comput. Biol.* **2017**, *13*, e1007081, doi:10.1101/117101.

22. Zhao, Q., Wang, J., Hemani, G., Bowden, J., & Small, D.S. Statistical Inference in Two-Sample Summary-Data Mendelian Randomization Using Robust Adjusted Profile Score. *Ann. Stat.* **2020**, *48*, 1742–1769.

23. Huang, D.W.; Sherman, B.T.; Stephens, R.; Baseler, M.W.; Lane, H.C.; Lempicki, R.A. DAVID Gene ID Conversion Tool. *Bioinformatician.Net* **2008**, *2*, 428–430.

24. Huang, D.W.; Sherman, B.T.; Lempicki, R.A. Systematic and Integrative Analysis of Large Gene Lists Using DAVID Bioinformatics Resources. *Nat. Protoc.* **2009**, *4*, 44–57, doi:10.1038/nprot.2008.211.

25. Huang, D.W.; Sherman, B.T.; Lempicki, R.A. Bioinformatics Enrichment Tools: Paths toward the Comprehensive Functional Analysis of Large Gene Lists. *Nucleic Acids Res.* **2009**, *37*, 1–13, doi:10.1093/nar/gkn923.

26. Brionne, A.; Juanchich, A.; Christelle, H.-A. An Overview of ViSEAGO: Visualisation, Semantic Similarity, Enrichment Analysis of Gene Ontology. Available online: http://bioconductor.org/packages/devel/bioc/vignettes/ViSEAGO/inst/doc/ViSEAGO.html.

27. Law, C.W.; Chen, Y.; Shi, W.; Smyth, G.K. Voom: Precision Weights Unlock Linear Model Analysis Tools for RNA-Seq Read Counts. *Genome Biol.* **2014**, *15*, R29, doi:10.1186/gb-2014-15-2-r29.

28. Liu, R.; Holik, A.Z.; Su, S.; Jansz, N.; Chen, K.; Leong, H.S.; Blewitt, M.E.; Asselin-Labat, M.L.; Smyth, G.K.; Ritchie, M.E. Why Weight? Modelling Sample and Observational Level Variability Improves Power in RNA-Seq Analyses. *Nucleic Acids Res.* **2015**, *43*, e97, doi:10.1093/nar/gkv412.

29. Levin, A.; Reznichenko, A.; Witasp, A.; Liu, P.; Greasley, P.J.; Sorrentino, A.; Bruchfeld, A.; Barany, P.; Blondal, T.; Zambrano, S.; et al. Novel Insights into the Disease Transcriptome of Human Diabetic Glomeruli and Tubulointerstitium. *Nephrol. Dial. Transplant.* **2020**, *35*, 2059–2072, doi:10.1093/ndt/gfaa121.

30. Fan, Y.; Yi, Z.; Agati, V.D.D.; Sun, Z.; Zhong, F.; Zhang, W.; Wen, J.; Zhou, T.; Li, Z.; He, L.; et al. Comparison of Kidney Transcriptomic Profiles of Early and Advanced Diabetic Nephropathy Reveals Potential New Mechanisms for Disease Progression. *Diabetes* **2019**, *68*, 2301–2314, doi:10.2337/db19-0204.

31. Afgan, E.; Baker, D.; Batut, B.; Van Den Beek, M.; Bouvier, D.; Ech, M.; Chilton, J.; Clements, D.; Coraor, N.; Grüning, B.A.; et al. The Galaxy Platform for Accessible, Reproducible and Collaborative Biomedical Analyses: 2018 Update. *Nucleic Acids Res.* **2018**, *46*, W537–W544, doi:10.1093/nar/gky379.

32. Benjamini, Y.; Hochberg, Y. Controlling the False Discovery Rate : A Practical and Powerful Approach to Multiple Testing. *J. R. Stat. Soc.* **1995**, *57*, 289–300.

33. Hadley Wickham *Ggplot2: Elegant Graphics for Data Analysis*; Springer-Verlag New York, 2016;
